# *In Vitro* Manganese Exposure Disrupts MAPK Signaling Pathways in Striatal and Hippocampal Slices from Immature Rats

**DOI:** 10.1155/2013/769295

**Published:** 2013-11-13

**Authors:** Tanara Vieira Peres, Daniela Zótico Pedro, Fabiano Mendes de Cordova, Mark William Lopes, Filipe Marques Gonçalves, Cláudia Beatriz Nedel Mendes-de-Aguiar, Roger Walz, Marcelo Farina, Michael Aschner, Rodrigo Bainy Leal

**Affiliations:** ^1^Departamento de Bioquímica, Centro de Ciências Biológicas, Universidade Federal de Santa Catarina, Campus Universitário, 88040-900 Florianópolis, SC, Brazil; ^2^Escola de Medicina Veterinária e Zootecnia, Universidade Federal do Tocantins, 77804-970 Araguaína, TO, Brazil; ^3^Departamento de Biologia Celular, Embriologia e Genética, Centro de Ciências Biológicas, Universidade Federal de Santa Catarina, Campus Universitário, 88040-900 Florianópolis, SC, Brazil; ^4^Departamento de Clínica Médica, Centro de Ciências da Saúde, Universidade Federal de Santa Catarina, Hospital Universitário (HU), 88036-800 Florianópolis, SC, Brazil; ^5^Department of Pediatrics, Vanderbilt University Medical Center, Nashville, TN 37232, USA

## Abstract

The molecular mechanisms mediating manganese (Mn)-induced neurotoxicity, particularly in the immature central nervous system, have yet to be completely understood. In this study, we investigated whether mitogen-activated protein kinases (MAPKs) and tyrosine hydroxylase (TH) could represent potential targets of Mn in striatal and hippocampal slices obtained from immature rats (14 days old). The aim of this study was to evaluate if the MAPK pathways are modulated after subtoxic Mn exposure, which do not significantly affect cell viability. The concentrations of manganese chloride (MnCl_2_; 10–1,000 **μ**M) caused no change in cell viability in slices exposed for 3 or 6 hours. However, Mn exposure significantly increased extracellular signal-regulated kinase (ERK) 1/2, as well as c-Jun N-terminal kinase (JNK) 1/2/3 phosphorylation at both 3 and 6 hours incubations, in both brain structures. Furthermore, Mn exposure did not change the total content or phosphorylation of TH at the serine 40 site in striatal slices. Thus, Mn at concentrations that do not disrupt cell viability causes activation of MAPKs (ERK1/2 and JNK1/2/3) in immature hippocampal and striatal slices. These findings suggest that altered intracellular MAPKs signaling pathways may represent an early event concerning the effects of Mn in the immature brain.

## 1. **Introduction**

Manganese (Mn) is an essential metal for humans and animals. Its essentiality is due to its requirement as a cofactor in chemical reactions catalyzed by several enzymes involved in cellular homeostasis, such as mitochondrial superoxide dismutase and glutamine synthetase. However, exposure to high Mn levels may cause a neurological syndrome referred to as manganism, which shares multiple analogous symptoms with Parkinson's disease (PD) [1]. 

Within the central nervous system (CNS), Mn accumulates predominantly in structures of the basal ganglia, such as the striatum (caudate, putamen, and nucleus accumbens), globus pallidus, and substantia nigra, consistent with dystonia, bradykinesia and rigidity secondary to damage to dopaminergic neurons and gliosis [2, 3]. Nonmotor symptoms have also been reported in Mn-exposed individuals, such as loss of attention and neuropsychological symptoms, suggesting that not only the basal ganglia are affected in manganism [1, 4]. 

Neonatal and developing brains are more susceptible to Mn toxicity. The uptake of metal in the intestine is usually elevated in the first week of life, followed by a steady decrease with age. These factors contribute to the increased risk for neurotoxicity in newborns when exposed to excess Mn [5]. In addition, increased hair Mn levels (a useful analytical biomarker of Mn exposure in humans) has been associated with learning disabilities, hyperactivity, and attention deficit disorder [5]. In rats, exposure to Mn during development has been shown to affect the dopaminergic system and induce lasting motor impairment and astrocyte activation [6]. Nevertheless, despite evidence suggesting that precocious Mn exposure may impair neurological function later in life [6, 7], studies on the molecular targets of Mn during critical periods of brain development have yet to be systematically addressed. 

Although the molecular mechanisms that mediate Mn-induced neurotoxicity have yet to be fully understood, evidence points to mitochondrial dysfunction with formation of reactive oxygen species (ROS) and oxidative stress as critical mediators of neurotoxicity [8–11]. Increased striatal levels of F2 isoprostanes as consequence of oxidative stress have been reported in response to Mn treatment in rats during development, with concomitant activation of the mitogen-activated protein kinase (MAPK) ERK1/2 [12, 13]. Of particular importance, studies in cell culture models have shown that Mn-induced oxidative stress is related, at least in part, to the activation of MAPK pathways [14–17]. MAPKs are a family of serine-threonine kinases which play critical roles in neurodevelopment and neuroplasticity. They mediate various cellular responses, such as proliferation, differentiation, cell survival, death, and cell transformation. The three major MAPKs are extracellular signal-regulated kinases (ERK1/2), c-Jun N-terminal kinases (JNKs), and p38^MAPK^ [18, 19]. 

Neurotransmission dyshomeostasis has been implicated in Mn neurotoxicity, and deregulated dopamine (DA) signaling has been a major focus of research (reviewed by Aschner et al. [1]). Due to similarities between manganism and PD and the accumulation of Mn in regions rich in dopaminergic (DAergic) neurons, altered DA metabolism has been considered an important aspect of the molecular actions of Mn [20]. Motor disturbances have been observed in children undergoing prolonged parenteral nutrition, where Mn is present at high concentrations [21]. *In vitro* evidence has shown that Mn decreases DA levels in the striatum due to direct oxidation of this monoamine [22]. Furthermore, Posser et al. [23] demonstrated that in PC12 cells low levels of Mn can induce sustained phosphorylation at serine (Ser) 40 of tyrosine hydroxylase (TH), the rate limiting enzyme for the synthesis of DA, leading to prolonged activation of the enzyme. 

As noted above, activation of MAPK pathways has been linked to Mn-induced neurotoxicity [12, 14, 15, 17]. However, it is difficult to affirm whether MAPK activation is a cause or consequence of Mn-induced neurotoxicity. No reports were located to ascertain whether MAPK activation represents a primary event (that could trigger Mn toxicity) or if it occurs secondary to Mn-induced cellular dyshomeostasis/damage (triggered via other mechanisms, i.e., oxidative or metabolic changes). Accordingly, the aim of this study was to evaluate if MAPK pathways are modulated after Mn exposure when this metal is present at *subtoxic* concentrations, which are unable to change cell viability. In addition, the potential activation of TH was also investigated since it is important in maintaining homeostasis in DAergic neurons (targeted by Mn). To meet these objectives, fresh striatal and hippocampal slices from rats in a critical neurodevelopmental period (postnatal day 14/PND14) were acutely exposed to Mn (for 3–6 h). Our study indicates that disturbances in ERK and JNK activity in response to Mn may represent an early event. The occurrence of changes in ERK activity and JNK activity after Mn exposure, added to the absence of cell viability loss, suggests that the modulation of MAPK signaling pathway represents a primary event induced by Mn.

## 2. Methods

### 2.1. Chemicals

Anti-phospho-ERK1/2, anti-phospho-JNK1/2/3 antibodies, and LumiGLO chemiluminescent substrate were purchased from Cell Signaling (Beverly, MA, USA). Anti-phospho-p38^MAPK^, anti-total-p38^MAPK^, anti-total-ERK1/2, and anti-total-JNK1/2 antibodies and manganese chloride (MnCl_2_) and Dulbecco's modified Eagle's medium (DMEM) were obtained from Sigma (St. Louis, MO, USA). Anti-phospho-TH, anti-total-TH, and goat anti-rabbit IgG HRP (horseradish peroxidase) conjugate secondary antibodies were purchased from Millipore (Billerica, MA, USA). Tris and *β*-mercaptoethanol were obtained from Amresco (Solon, OH, USA). 3-(4,5-dimethylthiazol-. 2-yl)-2,5-diphenyl tetrazolium bromide (MTT), SDS, and bis-acrylamide were from USB (Cleveland, OH, USA). Acrylamide and Hybond nitrocellulose were purchased from GE Healthcare Life Sciences (Piscataway, NJ, USA). All other reagents were of the highest analytical grade.

### 2.2. Animals

All animal studies were carried out in accordance with the “Principles of Laboratory Animal Care” (NIH publication number 80-23, revised 1996) and approved by the local Ethical Committee for Animal Research. Wistar rats of both genders at postnatal day 14 (PND14) were obtained from our own breeding colony at the Universidade Federal de Santa Catarina (UFSC), Brazil. At this developmental stage (PND14), female and male rats do not display differences in hormonal activity, since sexual maturity is not yet reached [24]. Rats were maintained in an air-conditioned room (22–25°C) on a 12-h light/dark cycle with water and food *ad libitum*. 

### 2.3. Striatal and Hippocampal Slice Preparation and Treatments

Preparation and treatment of striatal and hippocampal slices were performed as previously described [25–27]. Briefly, a total of 32 animals were euthanized by decapitation; their encephala were extracted and the striata and hippocampi were immediately dissected (4°C) in HEPES-saline buffer (124 mM NaCl, 4 mM KCl, 1.2 mM MgSO_4_, 12 mM D-glucose, 1 mM CaCl_2_, and 25 mM HEPES; pH 7.4), which was previously oxygenated for 30 min. Slices of 400 *μ*m thickness were prepared using a McIlwain Tissue Chopper. In order to obtain a sufficient number of slices, two immature rats (PND14) were used for each of the experiments. The hippocampal slices obtained were pooled and subsequently three slices for each treatment were carefully separated with a brush. The slices were individually preincubated with HEPES-saline buffer (300 *μ*L/slice) for 30 min at room temperature (RT). For the treatments, the HEPES-saline buffer was replaced by fresh buffer in the absence (control) or presence of MnCl_2_ (1–1,000 *μ*M) and incubated for 3 h at 37°C. These concentrations were chosen based on previous reports, which have used similar concentrations *in vitro* [22]. The concentrations chosen encompass the physiological (10 *μ*M), threshold of toxicity (100 *μ*M), and toxic (1,000 *μ*M) spectrum based on reports by Suzuki et al. [28] performed in monkeys.

The extended incubations (6 h) required different buffers similar to the conditions used for maintenance of organotypic slice cultures. The maintenance of cell viability under this experimental condition has been previously described [27]. Dissection (4°C) and preincubation (30 min, RT) were performed in Krebs-Ringer bicarbonate buffer (KRB) (122 mM NaCl, 3 mM KCl, 1.2 mM MgSO_4_, 1.3 mM CaCl_2_, 0.4 mM KH_2_PO_4_, 25 mM NaHCO_3_, and 10 mM D-glucose). The buffer was bubbled with 95% O_2_–5% CO_2_ to pH 7.4. For the treatments, the medium was replaced by a nutritive incubation medium composed of 50% KRB, 50% Dulbecco's modified Eagle's medium (DMEM), 20 mM of HEPES, and 100 *μ*g/mL of gentamicin, at 37°C in a 95% O_2_/5% CO_2_ atmosphere, in the absence (control) or presence of MnCl_2_ (1–1,000 *μ*M), and the slices were incubated for 6 h [26, 27].

### 2.4. Cell Viability

Slice viability was analyzed by the colorimetric MTT [3′-(4,5-dimethylthiazol-2yl) 2,5-diphenyltetrazolium bromide] reduction assay [29]. At the end of the treatments, the medium was removed and the slices (in triplicates) were incubated with 0.5 mg/mL MTT (200 *μ*L) in HEPES-saline buffer (in the case of 3 h treatments) or KRB (6 h treatments). The slices were incubated for 20 minutes at 37°C. Mitochondrial dehydrogenases in viable cells reduce MTT to formazan crystals, which are dissolved in dimethylsulfoxide (DMSO). The absorbance was quantified spectrophotometrically using a microplate reader (*λ* = 540 nm). Results are expressed as a percentage of the control (absence of MnCl_2_).

### 2.5. Western Blotting Analysis

To access MAPK activation, western blot analysis of slices samples was performed as previously described [25, 30, 31]. Briefly, the slices were solubilized with SDS-stopping solution (4% SDS, 2 mM EDTA, 50 mM Tris, 5% *β*-mercaptoethanol, pH 6.8) for 5 minutes at 100°C. Aliquots for protein concentration determination were collected before *β*-mercaptoethanol addition and protein concentration was later determined by the Peterson method [32]. Next, 25 : 100 (v/v) of sample dilution was added (40% glicerol, 25 mM Tris, bromophenol blue, and pH 6.8). Sixty *μ*g of total protein/track were separated by sodium dodecyl sulfate polyacrylamide gel electrophoresis (SDS-PAGE) using 10% gels (miniVE Vertical Electrophoresis System, GE Healthcare Life Sciences, Piscataway, NJ, USA), followed by transfer to nitrocellulose membranes using a semidry blotting apparatus (TE 70 SemiPhor Unit, GE Healthcare Life Sciences, Piscataway, NJ, USA) (1.2 mA/cm^2^; 1 h 30 min) as described by Bjerrum and Heegaard (1988). After blocking with 5% skim milk in Tris buffered saline (TBS) (Tris 10 mM, NaCl 150 mM, pH 7.5) for 1 hour, membranes were incubated overnight (4°C) with primary antibodies to detect the phosphorylated forms of ERK1/2, JNK1/2/3, p38^MAPK^, and TH, in the dilutions recommended by the manufacturer. Subsequently, membranes were incubated for 1 h at RT with anti-Rabbit IgG HRP conjugate secondary antibodies. All steps were followed by three 5 minutes washing with TBS-T (TBS with the addition of Tween-20 0.1% and pH 7.5). The blots were developed by chemiluminescent reaction. Subsequently, membranes were stripped of the antibodies using a solution of NaOH 0.2 N and then reprobed to detect the total forms (phosphorylated plus nonphosphorylated form) of the target proteins. The bands were quantified using the Scion Image software (Frederick, MD, USA). The ratio of the optic density (OD) of the phosphorylated protein band over the OD of the total protein band was calculated and the phosphorylation level of each protein was determined as a percentage of the control (considered 100%).

The antibody against ERK1/2 detected two bands, one at approximately 44 kDa and the second at approximately 42 kDa, corresponding, respectively, to the two ERK isoforms, ERK1 and ERK2. Anti-p38^MAPK^ detected a single band of approximately 38 kDa; anti-JNK 1/2/3 detected two bands, one at approximately 54 kDa and the second at approximately 46 kDa, corresponding, respectively, to the three JNK isoforms, JNK2/3 and JNK1.

### 2.6. Statistical Analyses

Statistical analyses were performed by one-way analysis of variance (ANOVA) followed by Duncan's *post hoc* test when appropriate using STATISTICA 5.1 software (SS Inc., Tulsa, OK, USA). Differences were considered to be significant when *P* < 0.05. The values were expressed as mean ± S.E.M.

## 3. Results

### 3.1. Assessment of Cell Viability in Striatal and Hippocampal Slices Exposed to Mn

**Figure 1:**
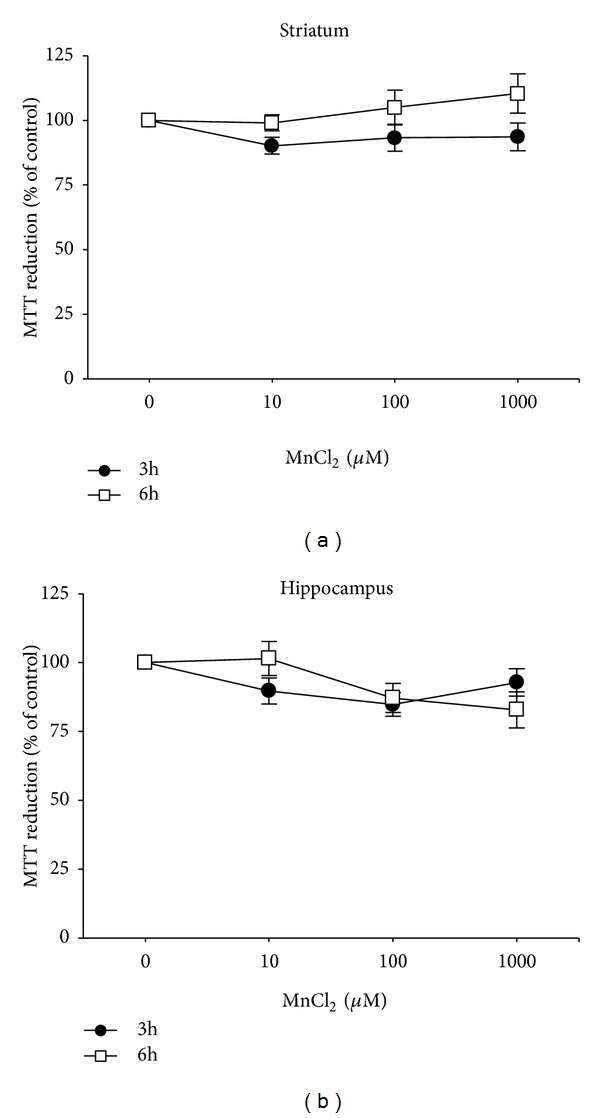
Effect of MnCl_2_ on cell viability in striatal (a) and hippocampal (b) slices from immature rats. Slices were incubated for 3 h or 6 h in the absence (control) or presence of MnCl_2_ (10–1000 *μ*M). Cell viability was assessed by analysis of MTT reduction. The data represent the percentage of MTT reduction compared to control (considered as 100%) and express the mean ± SEM derived from 4 independent experiments.

Previous studies show selective neurotoxicity of Mn toward basal ganglia structures, such as the striatum (caudate, putamen, and nucleus accumbens), globus pallidus, and substantia nigra [1]. In this study, *in vitro* Mn exposure (10–1,000 *μ*M) for 3 or 6 hours did not significantly affect cell viability in either striatal (Figure 1(a)) or hippocampal slices (Figure 1(b)) assessed by MTT. The results for 3 and 6 hours incubations were obtained separately and are expressed as percentage relative to the control.

### 3.2. MAPKs Modulation in Striatal Slices Exposed to Mn

The phosphorylation levels of ERK1/2, JNK1/2/3, and p38^MAPK^ were assessed in striatal or hippocampal slices isolated from immature rats (PND14) in response to *in vitro* Mn exposure. Slices were exposed for 3 or 6 h to Mn at concentrations ranging from 10–1,000 *μ*M and then analyzed by western blotting.

**Figure 2:**
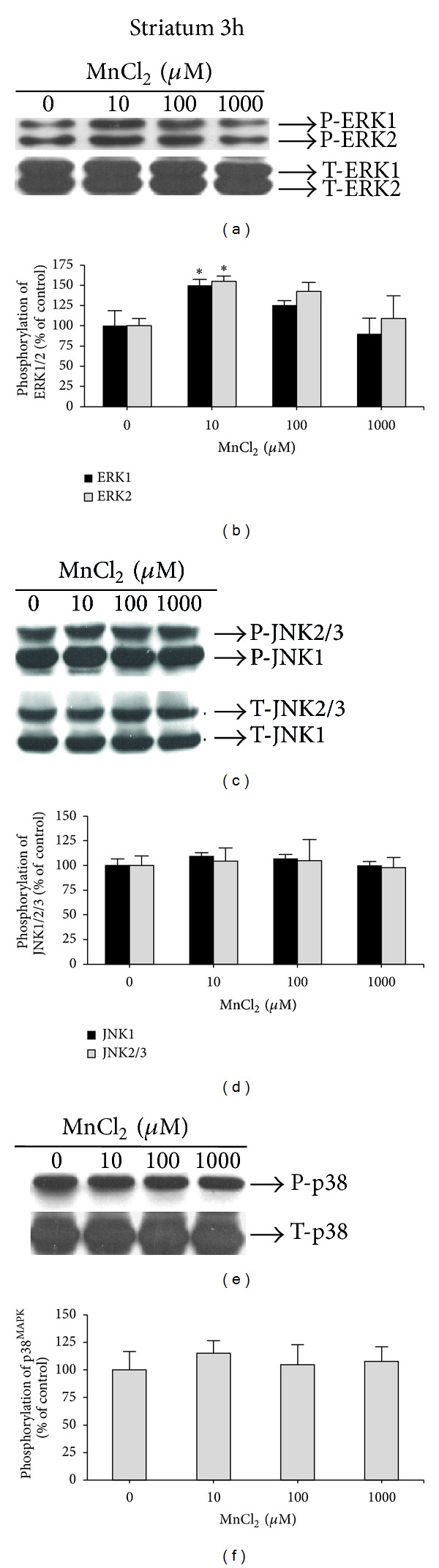
Effect of MnCl_2_ on MAPKs phosphorylation (ERK1/2, JNK1/2/3 and p38^MAPK^) in striatal slices from immature rats (PND14). Slices were incubated for 3 h in the absence (control) or presence of MnCl_2_ (10–1000 *μ*M). The panels (a), (c), and (e) show a representative blot of striatal immunoreactivity of the phospho (P)-ERK; and total (T)-ERK, P-JNK, and T-JNK; and P-p38^MAPK^; and T-p38^MAPK^, respectively. The panels (b), (d), and (f) show the striatal quantification of P-ERK, P-JNK, and P-p38^MAPK^, respectively. The phosphorylation level of each protein was determined by computer-assisted densitometry as a ratio of the O.D. of the phosphorylated (P) band over the O.D. of the total (T) band, and the data are expressed as percentage of the control. The values are presented as mean ± S.E.M derived from 4 independent experiments. *P < 0.05.

 Figures 2(a) and 2(b) show that Mn exposure (10 *μ*M, 3 h) significantly increased ERK1 and ERK2 phosphorylation (49.81 ± 7.66%) (*F*(3,12) = 3.53, *P* = 0.048) and 54.96 ± 6.43% (*F*(3,12) = 3.64, *P* = 0.045), respectively, in striatal slices. JNK1/2/3 (Figures 2(c) and 2(d)), and p38^MAPK^ (Figures 2(e) and 2(f)) phosphorylation were not significantly different from controls after 3 h exposure to Mn. 

**Figure 3:**
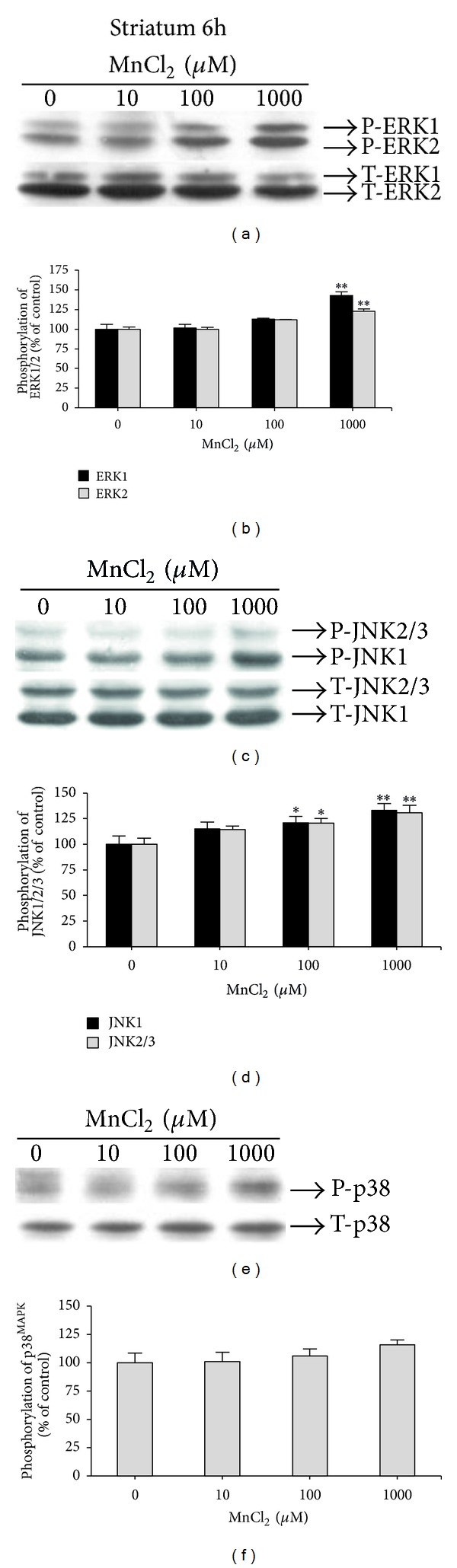
Effect of MnCl_2_ on MAPKs phosphorylation (ERK1/2, JNK1/2/3, and p38^MAPK^) in striatal slices from immature rats (PND14). Slices were incubated for 6 h in the absence (control) or presence of MnCl_2_ (10–1000 *μ*M). The panels (a), (c), and (e) show a representative blot of striatal immunoreactivity of the phospho (P)-ERK; and total (T)-ERK, P-JNK, and T-JNK; and P-p38^MAPK^; and T-p38^MAPK^, respectively. The panels (b), (d), and (f) show the striatal quantification of P-ERK, P-JNK, and P-p38^MAPK^, respectively. The phosphorylation level of each protein was determined by computer-assisted densitometry as a ratio of the O.D. of the phosphorylated (P) band over the O.D. of the total (T) band, and the data are expressed as percentage of the control. The values are presented as mean ± S.E.M derived from 4 independent experiments. **P < 0.01 and *P < 0.05.

When striatal slices were exposed to 1,000 *μ*M Mn for 6 h, significant increases in ERK1 (42.96 ± 4.48%) (*F*(3,12) = 19.79, *P* = 0.000465), ERK2 (22.65 ± 2.84%) (*F*(3,12) = 23.25, *P* = 0.000264) (Figures 3(a) and 3(b)), JNK1 (33.29 ± 6.60%) (*F*(3,19) = 3.88, *P* = 0.025), and JNK2/3 (30.65 ± 7.29%) (*F*(3,19) = 5.11, *P* = 0.00922) (Figure 3(d)) phosphorylation were observed. However, only JNK1 (21.00 ± 6.16%) (*F*(3,19) = 3.88  *P* = 0.025) and JNK2/3 (20.65 ± 4.59%) (*F*(3,19) = 5.11, *P* = 0.00922) phosphorylation (Figures 3(c) and 3(d)) were significantly increased after exposures to 100 *μ*M Mn and no significant changes were observed in p38^MAPK^ phosphorylation under the same experimental conditions (Figures 3(e) and 3(f)).

### 3.3. MAPKs Modulation in Hippocampal Slices Exposed to Mn

**Figure 4:**
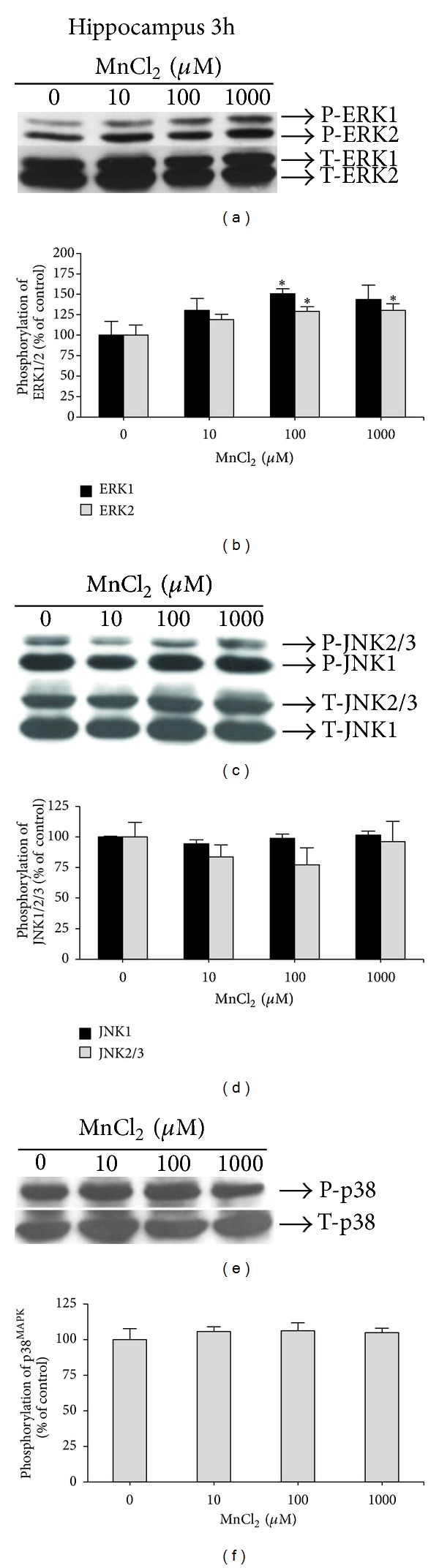
Effect of MnCl_2_ on MAPKs phosphorylation (ERK1/2, JNK1/2/3, and p38^MAPK^) in hippocampal slices from immature rats (PND14). Slices were incubated for 3 h in the absence (control) or presence of MnCl_2_ (10–1000 *μ*M). The panels (a), (c), and (e) show a representative blot of hippocampal immunoreactivity of the phospho (P)-ERK; and total (T)-ERK, P-JNK, and T-JNK, and P-p38^MAPK^; and T-p38^MAPK^, respectively. The panels (b), (d), and (f) show the hippocampal quantification of P-ERK, P-JNK, and P-p38^MAPK^, respectively. The phosphorylation level of each protein was determined by computer-assisted densitometry as a ratio of the O.D. of the phosphorylated (P) band over the O.D. of the total (T) band, and the data are expressed as percentage of the control. The values are presented as mean ± S.E.M derived from 4 independent experiments. *P < 0.05.

Hippocampal slices exposed to Mn for 3 h showed a significant increase of ERK1 (50.44 ± 6.47%) (*F*(3,19) = 3.23, *P* = 0.046) and ERK2 phosphorylation (29.02 ± 5.45%) (*F*(3,19) = 3.48, *P* = 0.042) at 100 *μ*M Mn (Figures 4(a) and 4(b)). Moreover, ERK2 phosphorylation was also significantly increased (30.62 ± 7.80%) (*F*(3,19) = 3.48, *P* = 0.042) at the highest Mn concentration tested (1,000 *μ*M). JNK1/2/3 (Figures 4(c) and 4(d)) and p38^MAPK^ (Figures 4(e) and 4(f)) phosphorylation were not modified in the hippocampal slices after 3 h exposure to Mn.

**Figure 5:**
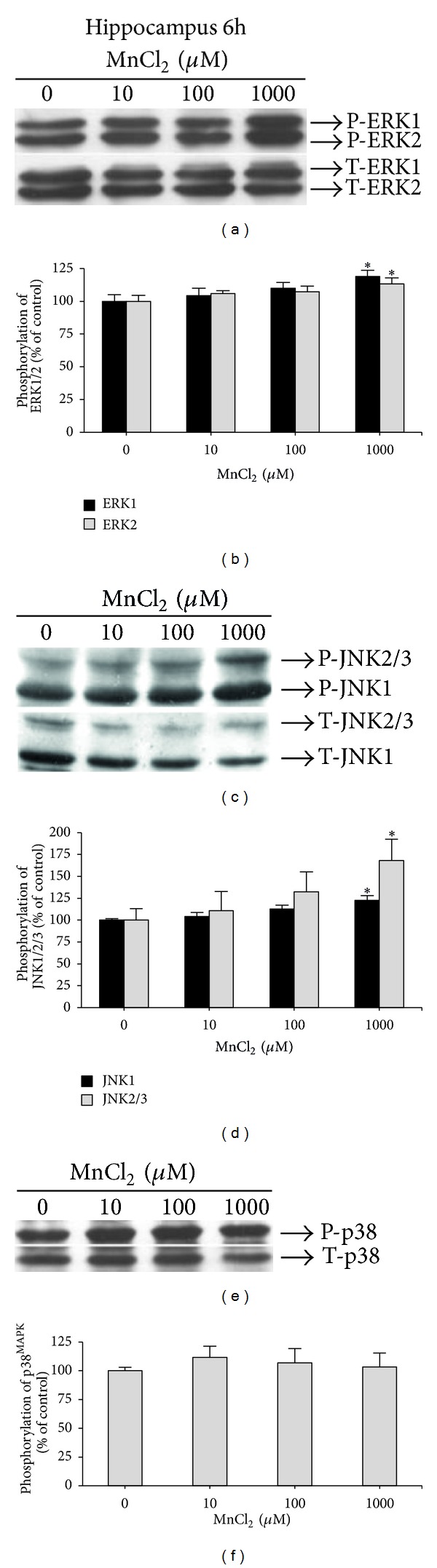
Effect of MnCl_2_ on MAPKs phosphorylation (ERK1/2, JNK1/2/3, and p38^MAPK^) in hippocampal slices from immature rats (PND14). Slices were incubated for 6 h in the absence (control) or presence of MnCl_2_ (10–1000 *μ*M). The panels (a), (c), and (e) show a representative blot of hippocampal immunoreactivity of the phospho (P)-ERK; and total (T)-ERK, P-JNK, and T-JNK; and P-p38^MAPK^; and T-p38^MAPK^, respectively. The panels (b), (d), and (f) show the hippocampal quantification of P-ERK, P-JNK, and P-p38^MAPK^, respectively. The phosphorylation level of each protein was determined by computer-assisted densitometry as a ratio of the O.D. of the phosphorylated (P) band over the O.D. of the total (T) band, and the data are expressed as percentage of the control. The values are presented as mean ± S.E.M derived from 4 independent experiments. *P < 0.05.

When hippocampal slices were exposed to Mn for 6 h, a significant increase in the phosphorylation of ERK1 (19.04 ± 4.48%) (*F*(3,19) = 3.21, *P* = 0.046) and ERK2 (13.34 ± 4.46%) (*F*(3,19) = 3.02, *P* = 0.049) (Figures 5(a) and 5(b)), JNK1 (22.58 ± 5.41%) (*F*(3,19) = 5.22, *P* = 0.008), and JNK2/3 (67.97 ± 24.94%) (*F*(3,19) = 3.11, *P* = 0.048) (Figures 5(c) and 5(d)) were observed at the highest Mn concentration (1,000 *μ*M). No significant changes were observed in p38^MAPK^ phosphorylation under the same experimental conditions (Figures 5(e) and 5(f)).

### 3.4. Phosphorylation of TH at Ser^40^ in Striatal Slices Exposed to Mn

**Figure 6:**
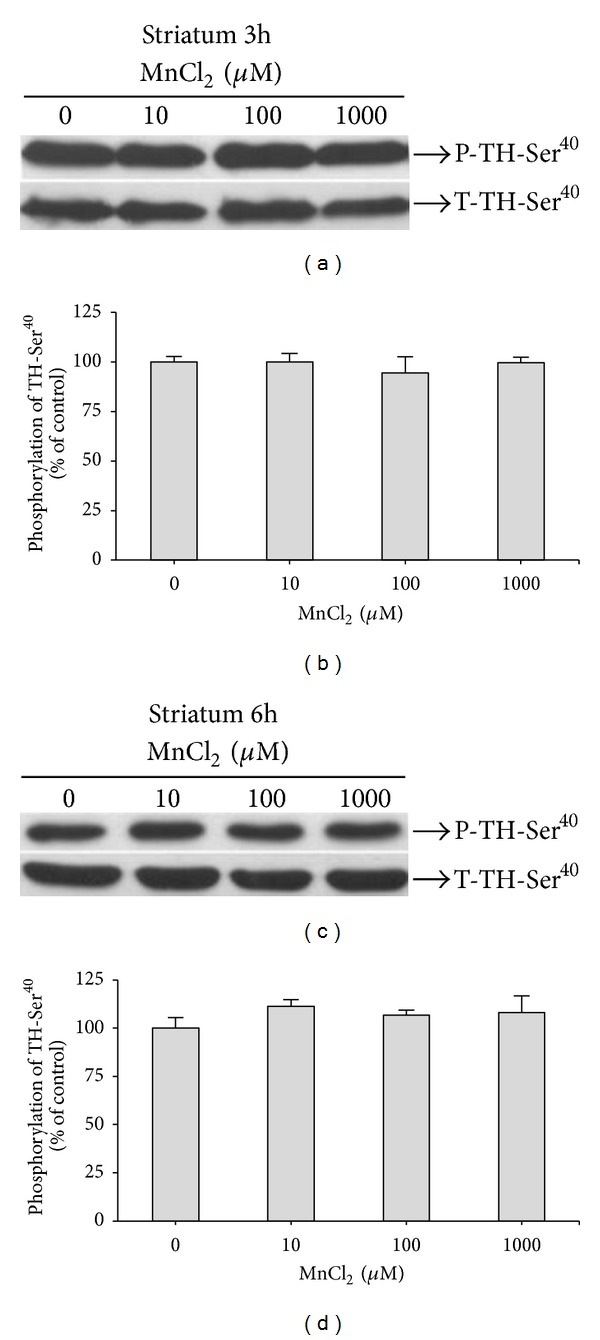
Effect of MnCl_2_ on phosphorylation of tyrosine hydroxylase at serine 40 (TH-Ser^40^) in striatal slices from immature rats (PND14). Slices were incubated for 3 and 6 h in the absence (control) or presence of MnCl_2_ (10–1000 *μ*M). The panel (a) shows a representative blot of striatal immunoreactivity of the phospho (P)-TH-Ser^40^ and total (T)-TH of the slices exposed for 3 h. The panel (b) shows the striatal quantification of P-TH-Ser^40^ of the slices exposed for 3 h. The panel (c) shows a representative blot of striatal immunoreactivity of the P-TH-Ser^40^ and T-TH of the slices exposed for 6 h. The panel (d) shows the striatal quantification of P-TH-Ser^40^ of the slices exposed for 6 h. The phosphorylation level of each protein was determined by computer-assisted densitometry as a ratio of the O.D. of the phosphorylated (P) band over the O.D. of the total (T) band, and the data are expressed as percentage of the control. The values are presented as mean ± S.E.M derived from 4 independent experiments.

TH is the rate-limiting enzyme for dopamine (DA) synthesis. Medium- and long-term modulation of TH activity occurs by regulation of gene expression and in the short-term by regulation of enzyme activity. Phosphorylation on serine residues, by several enzymes, is the primary mechanism of short-term TH activity regulation [33, 34]. Previous studies in PC12 cells demonstrated increased Ser^40^ phosphorylation and activation of TH in response to Mn [23]. However, in our experimental protocol (with tissue slices) no significant changes in TH-Ser^40^ phosphorylation in response to Mn (10–1,000 *μ*M) exposure were observed (Figure 6).

## 4. Discussion

Recent data from our group demonstrated that developmental Mn exposure from postnatal days 8–12 (PND8–12) altered the activity of key cell signaling elements, causing increased phosphorylation of DARPP-32-Thr-34, ERK1/2, and AKT in the striatum of rats on PND14 [12]. Additionally, in the same experimental protocol, Mn impaired motor coordination later at the 3rd, 4th, and 5th week of life. These results were in line with recent evidence indicating that MAPK pathways might be involved in the neurotoxicity induced by Mn in various experimental models, such as mesencephalic cells [16], PC12 cells [15, 23, 35], microglial cells [36, 37], primary astrocyte culture [17], and *in vivo *developmental exposure [12, 13]. However, the current knowledge on Mn-induced neurotoxicity does not allow for stating whether changes in MAPK signaling pathways represent cause or consequence of Mn-induced cell toxicity. In an attempt to investigate if Mn can modulate MAPK signaling pathways in conditions where cells are still completely viable, we took advantage of a slice-based model to evaluate Mn-induced effects *in vitro* with particular emphasis on striatal and hippocampal tissues. Employing experimental conditions (Mn concentration and exposure time) that do not cause significant cell death (evaluated by the MTT assay; Figure 1), we observed that both striatal and hippocampal slices showed significant changes in MAPKs phosphorylation. 

Our results showed that slice incubation with MnCl_2_ did not induce significant changes in cell viability (Figure 1). This finding corroborates findings by Sistrunk et al. [22], who failed to observe Mn-induced cell death in an *in vitro* model of rat striatal slices exposed to MnCl_2_ (10–1,000 *μ*M) for 4 hours. However, a decrease in cell viability *in vitro* was demonstrated in DAergic PC12 cells [14, 23] after exposure to high Mn concentrations for extended periods (24–48 h). It needs to be taken into account that the cell viability in the present work was assessed only by the MTT test. Other methods such as lactate dehydrogenase (LDH) or neutral red could be tested in future works. Nevertheless, as mentioned above, it needs to be considered that studies in pure PC12 cultures are void of glial cells, which are known modulators of Mn CNS homeostasis. Astrocytes accumulate higher levels of Mn than neurons and may be an initial site for Mn-induced damage, thus, lowering the neuronal burden of Mn [8]. Furthermore, the slice model provides only a short time window for metal exposure (in our case a maximum of 6 hours) beyond which the cell viability is compromised [27]. Lead (Pb), another metal widely recognized as a neurotoxicant [38], has also been evaluated in the slices model [25]. No changes in cell viability were observed after the treatment of hippocampal slices from PND14 rats with Pb acetate. However, phosphorylation levels of ERK and p38^MAPK^ were increased. These results indicate that even though cell viability is not compromised, toxic metals may still cause changes in cells signaling pathways *in vitro* that may disturb cellular function [25].

Our results showed an increase in ERK phosphorylation in striatal slices exposed to a low Mn concentration (10 *μ*M) for 3 hours (Figure 2(b)). ERK and JNK phosphorylation also increased in striatal slices exposed to 100 and 1,000 *μ*M Mn for 6 hours (Figures 3(b) and 3(d), resp.). Of note, these changes in MAPK signaling pathways were observed in slices completely viable (no changes in the MTT assay), indicating that altered intracellular MAPKs signaling pathways may represent an early event of Mn action in the slices. ERK1/2 activation has also been observed in the striatum of immature rats (PND14) developmentally exposed to Mn (PND8–12) [12]. Therefore, it appears that activation of ERK1/2 by Mn may be an early and conserved mechanism observed both *in vitro* and *in vivo*. 

The majority of *in vitro* studies concerning the effects of Mn on cell signaling pathways were conducted in cell culture models [23, 39]. However, neuroglial interactions may play important roles in the modulation of neurotoxic processes, including those triggered by metals [40, 41]. Thus, *in vitro *studies, which in general are focused on single cell type (e.g., neuron or astrocyte alone), fail to take into account toxic events that are dependent upon neuronal and glial interactions. In contrast, tissue slices from rat brain maintain the natural extracellular matrix, neuronal connectivity, and neuroglial interactions, providing an appropriate experimental model for studies on acute neurotoxic events [42, 43]. The model of 400 *μ*m thick brain slices is well established [42, 44] and has been used for *in vitro* studies on the neurotoxicity of metals, such as cadmium (Cd) [30] and Pb [25].

Striatal slices are rich in DAergic neurons, in contrast to hippocampal slices. Of particular importance, Mn shows selective neurotoxicity to DAergic neurons by mechanisms that have yet to be fully defined but possibly involve interaction with DA, leading to its oxidation forming toxic quinones and consequent oxidative stress [45–47]. A recent study in *Caenorhabditis elegans* showed that extracellular and not intracellular DA is responsible for Mn-induced DAergic neurodegeneration [48]. Moreover, it is known that the striatum expresses higher levels of DMT-1, which is the predominant transporter for divalent Mn into cells [49]. Therefore, the presence of DAergic neurons and DMT1 in striatal slices may explain, at least in part, the significant modulation of MAPKs by a relative low Mn concentration in striatal tissue as compared to hippocampus. 

With respect to the susceptibility of hippocampal slices to Mn, we noted increased ERK1/2 phosphorylation following exposure of hippocampal slices to 100 and 1,000 *μ*M Mn for 3 hours (Figure 4(b)). ERK and JNK also showed increased phosphorylation in hippocampal slices exposed to 1,000 *μ*M Mn for 6 h (Figures 5(b) and 5(d), resp.). In a recent study, the hippocampus was studied as a site for Mn neurotoxicity *in vivo*, since Mn exposure increased the levels of thiobarbituric acid reactive species (TBARS), diminished total thiol content, and increased superoxide dismutase and catalase activity, indicating oxidative stress within this structure [50]. Therefore, although the basal ganglia are the most susceptible sites to Mn neurotoxicity, it is also evident that Mn may cause broad spectrum neuropathological changes in both neurons and glia in other brain regions, which may account for some of the nonmotor symptoms associated with this disorder [1].

Given the (1) importance of TH in controlling DA levels in neurons, (2) the involvement of DA metabolism in Mn neurotoxicity, as well as (3) a plethora of studies showing TH activity modulation and Ser^40^ phosphorylation by Mn [23, 39], we investigated Mn's effects on TH Ser^40^ phosphorylation in striatal slices from immature rats. This site is phosphorylated by protein kinase A (PKA) and protein kinase C (PKC), increasing TH activity. Ser^40^dephosphorylation by protein phosphatase 2A (PP2A) results in decreased TH activity [33]. Our results showed no significant alteration in TH phosphorylation at Ser^40^ (Figure 6). This result indicates that in immature striatal slices Mn does not act in an analogous way to PC12 cells exposed to Mn (100 *μ*M). In the latter study, a sustained TH-Ser^40^ phosphorylation (6–24 h) was observed, independent from H_2_O_2_ production and in the absence of significant changes in cell viability [23]. 

Thus, based on our and previous studies, it can be inferred that Mn requires longer exposure periods to induce cell death in slices. However, important molecular changes were observed within short periods after Mn exposure, such as the activation of ERK1/2 and JNK1/2/3 signaling pathway (Figures 2–5). These findings are in agreement with those previously reported by our group in a model of developmentally exposed rats where ERK1/2 activation but not JNK1/2/3 was noted [12]. Thus, striatal slices of immature rats exposed *in vitro* to Mn reproduce (at least in part) the changes observed *in vivo*.

## 5. Conclusions

A key finding in this study is the Mn-dependent activation of ERK1/2 and JNK1/2/3 in striatal and hippocampal slices with no significant changes in cell viability. This finding indicates that MAPKs activation may represent an important primary event that precedes the deleterious effects of Mn on the cellular viability. Extrapolating our data to *in vivo *conditions, one would posit that Mn is able to modulate the activation of MAPKs in tissues that maintain their metabolic competence and viability. The physiological significance of such activation represents a research topic that needs further attention.
